# RADAR – A randomised, multi-centre, prospective study comparing best medical treatment versus best medical treatment plus renal artery stenting in patients with haemodynamically relevant atherosclerotic renal artery stenosis

**DOI:** 10.1186/1745-6215-10-60

**Published:** 2009-07-27

**Authors:** Uwe Schwarzwälder, Michael Hauk, Thomas Zeller

**Affiliations:** 1 Department Angiology, Herz-Zentrum Bad Krozingen, Südring 15, 79189 Bad Krozingen, Germany

## Abstract

**Background:**

Prospective, international, multi-centre, randomised (1:1) trial to evaluate the clinical impact of percutaneous transluminal renal artery stenting (PTRAS) on the impaired renal function measured by the estimated glomerular filtration rate (eGFR) in patients with haemodynamically significant atherosclerotic renal artery stenosis.

**Methods:**

Patients will be randomised to receive either PTRAS using the Dynamic Renal Stent system plus best medical treatment or best medical treatment. Renal stenting will be performed under angiographic imaging. For patients randomised to best medical treatment the degree of stenosis measured by renal duplex sonography (RDS) will be confirmed by MR angio or multi-slice CT where possible. Best medical treatment will be initiated at randomisation or post procedure (for PTRAS arm only), and adjusted as needed at all visits. Best medical treatment is defined as optimal drug therapy for control of the major risk factors (blood pressure ≤ 125/80 mmHg, LDL cholesterol ≤ 100 mg/dL, HbA1c ≤ 6.5%). Data recordings include serum creatinine values, eGFR, brain natriuretic peptide, patients' medical history and concomitant medication, clinical events, quality of life questionnaire (SF-12v2™), 24 hour ambulatory blood pressure measurement, renal artery duplex ultrasound and echocardiography. Follow-up intervals are at 2, 6, 12 and 36 months following randomisation.

The primary endpoint is the difference between treatments in change of eGFR over 12 months. Major secondary endpoints are technical success, change of renal function based on the eGFR slope change between pre-treatment and post-treatment (i.e. improvement, stabilisation, failure), clinical events overall such as renal or cardiac death, stroke, myocardial infarction, hospitalisation for congestive heart failure, progressive renal insufficiency (i.e. need for dialysis), need of target vessel revascularisation or target lesion revascularisation, change in average systolic and diastolic blood pressure, change in left ventricular mass index calculated from echocardiography, difference in the size of kidney (pole to pole length) measured by renal duplex sonography, total number, drug name, drug class, daily dose, regimen and Defined Daily Dose (DDD), of anti-hypertensive drugs, and change in New York Heart Association (NYHA) classification. Approximately 30 centres in Europe and South America will enrol patients. Duration of enrolment is expected to be 12 months resulting in study duration of 48 months.

**Trial registration:**

**Trial registration number**: NCT00640406

## Background

Renal artery stenosis (RAS) usually refers to a disease of the large extra-renal arterial vessels and most frequently is caused by atherosclerotic obstructions. Arterial hypertension, progressive renal failure and flash pulmonary oedema are clinical manifestations of RAS requiring intervention and treatment and may be resolved by revascularisation therapy. In the past, RAS was under recognised, under diagnosed and under treated. With improved non-invasive imaging techniques such as magnetic resonance imaging angiography, computed tomography angiography and high resolution renal duplex sonography, the diagnosis is currently more frequently established.

Atherosclerosis accounts for approximately 90% of cases with RAS and most commonly involves the origin and the proximal third of the main renal artery. In fact, ostial RAS can be considered as a combined disease of the aorta and the renal artery, rather than an isolated problem of the renal arteries. The prevalence of atherosclerotic RAS increases with age, male gender, traditional cardiovascular risk factors (hypertension, diabetes, smoking, disease, in particular aorto-iliac occlusive disease) [1]. However, the true prevalence of atherosclerotic RAS in unselected patients is unknown. In hypertensive patients, a significant RAS is observed in below 5% [2], whereas a prevalence of up to 12% has been reported in patients with coronary artery disease undergoing cardiac catheterisation [3] and up to 40% in patients with peripheral artery disease (PAD). Undoubtedly, atherosclerotic RAS is a progressive disease [4], as more than half of the patients exhibit an increasing degree of stenosis within five years after diagnosis [5], and one out of five patients with a critical stenosis suffers renal atrophy and renal failure during this period [1].

RAS may be treated conservatively by so called best medical treatment, surgically, or by endovascular interventions using balloon angioplasty and stenting. Applying endovascular therapy, there is general agreement that ostial lesions should be stented as it improves primary patency rates and reduces the burden of reintervention [6-11].

The indications for endovascular treatment are a matter of ongoing debates. Curing hypertension by means of angioplasty rarely occurs, although the number of antihypertensive medication usually can be reduced after successful treatment. Currently three randomised controlled trials (RCT) on the effects of balloon angioplasty compared to medical treatment of RAS in hypertensive patients have been published [12-15], and a systematic review re-analysed the combined data [15]. However, there is still a lack of evidence concerning the indications for treatment in patients with hypertension, because each of these studies had severe limitations.

Targeting ischaemic nephropathy, according to single centre reports revascularisation could stabilise or at least slow the decline of renal function. However, none of the former RCT's was designed to analyse this clinical endpoint. The largest of these 3 trials – DRASTIC [12] – did not find any advantage of angioplasty with respect to renal function. Yet, interpretation of DRASTIC is hampered by a high proportion of cross-over (48%) from best medical therapy to intervention after 3 months of follow-up due to insufficient blood pressure control and an out-dated interventional technique (plain balloon angioplasty). Moreover, the study included patients with moderate diameter stenosis (50% to 70%) that are unlikely to cause haemodynamic compromise. Nevertheless, in the balloon group, an increase of GFR of 15 ml/min was documented whereas best medical therapy resulted in a slight decline of GFR. This result was insignificant as a consequence of small sample size; only 102 patients were enrolled in this trial.

Interventional treatment has been demonstrated to be safe, durable and effective at improving hypertension, slowing the progressive decline in renal function [12,16-20] and in cases also improving renal function [7,20]. Nevertheless, angioplasty also bears the risk of inducing renal deterioration. Careful patient selection remains the most crucial point in renal interventions; however, current data are insufficient to give final recommendations on this issue. Even the just recently orally presented results of the up to date largest randomised trial (ASTRAL – Angioplasty and STent for Renal Artery Lesions presented by lead investigator Philip Kalra at the annual meeting of the American College of Cardiology ACC 2008) are hampered by a large selection bias of the enrolled patients.

This study (RADAR) is designed to compare the best medical treatment versus the best medical treatment plus renal artery stenting with the Dynamic Renal stent (BIOTRONIK, Bülach, Switzerland) in patients with haemodynamically significant atherosclerotic renal artery stenosis. The key inclusion criterion is the proof of significance of RAS by duplex ultrasound in terms of significantly reduced intra-renal resistance index and extended acceleration time at the affected side. Both parameters correlate closely with an angiographic diameter reduction of at least 70%. This selection criterion should exclude patients without haemodynamically relevant RAS from study entry.

## Methods

### Study design

RADAR is designed as randomised, multi-centre, prospective study. Patients with haemodynamically relevant atherosclerotic RAS matching the inclusion and passing all exclusion criteria will be randomised to receive best medical treatment or best medical treatment plus renal artery stenting. Best medical treatment is defined as optimal drug therapy for control of hypertension (blood pressure ≤ 125/80 mmHg), of hypercholesterolemia (LDL ≤ 100 mg/dL), and of diabetes (HbA1c ≤ 6.5%).

**Figure 1:**
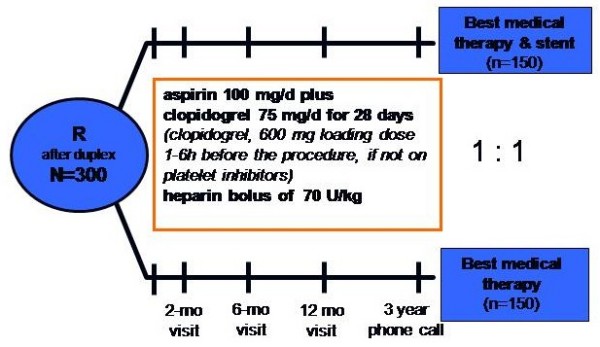
**Study profile**.

The objective of this study is to evaluate the clinical impact of percutaneous transluminal renal artery stenting (PTRAS) on the impaired renal function measured by the estimated Glomerular Filtration Rate (eGFR) in patients with haemodynamically relevant atherosclerotic RAS. Furthermore the effect of PTRAS on renal function, hypertension and quality of life in comparison to medical treatment will be evaluated. Safety and efficacy of both treatments are further endpoints of this study. Figure 1 shows the study profile.

Serum Creatinine (SCr) values and eGFR will be assessed from patient's medical files after obtaining informed consent, and a blood sample for determination of the baseline value will be obtained. At time of randomisation the patient's medical history and concomitant medication, clinical events and quality of life will be recorded. A 24 hour ambulatory blood pressure measurement will be obtained. Renal stenting will be performed under angiography imaging. For patients randomised to best medical treatment the degree of stenosis measured by renal duplex sonography will be confirmed by MR angiogram or multi-slice CT where possible. Best medical treatment will be initiated at randomisation or post procedurally (for PTRAS arm only), and adjusted as needed at all visits. For all patients renal duplex sonography will be performed at baseline, post procedure (for PTRAS arm only), and at 6 and 12 months follow up. Clinical events will be obtained at baseline and during all visits including a telephone follow up 3 years after randomisation. Twenty four hour ambulatory blood pressure measurement and blood sampling for eGFR determination will be done at baseline, post procedure (for PTRAS arm only), and after 2, 6 and 12 months. Adverse events and concomitant medication will be recorded up to 12 months after randomisation. At baseline, 6 and 12 months patients' quality of life will be assessed using a validated general health quality instrument (SF-12v2™). For further assessments see flow chart and table of events. Patients randomised to the 'no revascularisation'-group and presenting with a severe increase in serum creatinine concentration indicating the need of renal replacement therapy, or recurrent flash pulmonary oedema despite existing multidrug therapy (5 drugs), may receive a Dynamic Renal stent.

Only patients who have given written informed consent in accordance with the applicable regulations, the Declaration of Helsinki, GCP and ISO 14155 will be enrolled in the trial. The trial is conducted in compliance with the Declaration of Helsinki. The trial was approved by the central ethics commission "Ethik-Komission der Albert-Ludwigs-Universität Freiburg", Freiburg, Germany

### Endpoints

*Primary Endpoint*: The primary endpoint is the difference between treatments in change of eGFR over 12 months.

*Secondary Endpoints*: Secondary endpoints are defined as:

• Change in eGFR at 2, 6 and 12 months.

• Difference between treatments in change of eGFR over 2 and 6 months.

• Difference in eGFR slope between pre-treatment and post treatment time points at 2, 6 and 12 months.

• Change of renal function based on the eGFR slope change between pre-treatment and post-treatment at 2, 6 and 12 months (i.e. improvement, stabilisation, failure).

• Ratio of average Resistance Index (RI) at baseline, 6 and 12 months. Intra-renal Resistance Index is defined and calculated by the following formula: Intra-renal RI = (Peak Systolic Velocity-End Diastolic Velocity)/Peak Systolic Velocity.

• Degree of restenosis at 6 and 12 months.

• Clinical events overall: renal or cardiac death, stroke, myocardial infarction, hospitalisation for congestive heart failure, progressive renal insufficiency (i.e. need for dialysis), need for permanent renal replacement therapy, and need of target vessel revascularisation (TVR) or target lesion revascularisation (TLR) at 2, 6 and 12 months, and 3 years.

• Clinical events renal: renal death according to ICD 10, progressive renal insufficiency (i.e. need for dialysis), need for permanent renal replacement therapy, and need of TVR or TLR at 2, 6 and 12 months, and 3 years.

• MACCE: cardiac death, stroke, myocardial infarction, hospitalisation for congestive heart failure at 2, 6 and 12 months, and 3 years.

• Technical success, defined as successful access and deployment of the device with appropriate lesion coverage, stent positioning and patency determined by angiography.

• Acute procedural success, defined as residual percent diameter stenosis <30% by quantitative computerised angiography.

• Procedural success, defined as successful lesion crossing and stent positioning without the occurrence of a serious adverse event up to the moment the introducer sheath is removed,

• Average systolic and diastolic blood pressure (i.e. ambulatory 24 h-BP) at baseline, post procedure, 2, 6 and 12 months.

• Change in average systolic and diastolic blood pressure (i.e. ambulatory 24 h-blood pressure monitoring) at 2, 6 and 12 months.

• Left ventricular mass index (LVMI) calculated from echocardiography or cardiac magnet resonance imaging at baseline and 12 months. The LVMI [g/m^2^] is defined as left ventricular mass divided by body surface area [m^2^]. Left ventricular mass is calculated by using the method described by Devereux and Reichek [21], or as supplied by the manufacturer of the echo device.

• Difference in the size of kidney (pole to pole length) at baseline, 6 and 12 months measured by renal duplex sonography.

• Total number, drug name, drug class, daily dose, regimen and Defined Daily Dose (DDD), of anti-hypertensive drugs at baseline, 2, 6 and 12 months.

• Change in anti-hypertensive therapy at 2, 6 and 12 months.

• Classification of heart failure according to the New York Heart Association (NYHA) at baseline, 2, 6 and 12 months.

• Change in NYHA classification of heart failure at 2, 6 and 12 months.

• Quality of life at baseline, 6 and 12 months (i.e. SF-12v2™).

• Change in quality of life at 6 and 12 months.

• Patient survival

• Laboratory parameters at baseline, 2, 6 and 12 months (blood), or baseline and 12 months (urine).

• Adverse events.

### Patient population

Three hundred patients will be included to yield at least 250 patients with valid primary endpoint. Patients will be enrolled in approximately 30 centres in Europe and South America. Patient recruitment should be finished within 1 year following the initiation of the last study centre. Study duration would be 4 years.

Selection of participants for this study will be documented on the screening log. The patients being eligible for this study must meet ALL of the following inclusion and NONE of the exclusion criteria.

#### Inclusion Criteria

1. Age ≥ 18 years,

2. Informed consent signed by patient (and/or legal guardian),

3. Haemodynamically relevant de novo unilateral or bilateral RAS (≥ 70%). In case of unilateral disease, haemodynamically relevant RAS is defined by an intra-renal Resistance Index difference dRI > 0.05 (RI values comparison between the two individual kidneys). The lower RI value indicates the kidney with the stenosed artery. In presence of bilateral symmetric disease, RAS ≥ 70% is defined by an acceleration time more than 0.07 sec.

4. Estimated GFR > 10 ml/min calculated using the abbreviated Modification of Diet in Renal Disease (MDRD) Study equation,

5. Patients presenting mild, moderate or severe hypertension (defined according to the WHO guidelines) and/or renal dysfunction,

6. Target lesion must be completely coverable by one study stent (total target lesion length estimated to be less than 19 mm),

7. Target lesion accessible to direct stenting or, after pre-dilation, is likely to sufficiently benefit from stenting (at the discretion of the investigator),

8. Renal reference vessel diameter (RVD) of ≥ 4.0 mm and < 7.0 mm based on visual estimation,

9. Willingness to comply with all the specified follow-up evaluations.

#### Exclusion Criteria

Target indication and treatment:

1. Estimated GFR ≤ 10 ml/min,

2. Renal atrophy or kidney length < 7 cm (referring to kidney with target lesion),

3. Patient not eligible for PTRA,

4. Patient not eligible for stenting,

5. Target lesion occlusion,

6. Target lesion and/or target vessel proximal to the target lesion is severely calcified,

7. Treatment of branch lesion required,

8. Fresh thrombus or embolic lesion,

##### Medical history

9. Need for embolic protection in previous or planned PTRAS,

10. Clotting disorders,

11. INR < 2.5 in patients using warfarin treatment,

12. Patient presents fibromuscular dysplasia,

13. Prior revascularisation of target lesion,

14. History of target vessel revascularisation within the last 6 months,

15. Angiographic restenosis of any segment of the target vessel that has undergone prior percutaneous intervention,

16. Any thrombolytic therapy procedure within 72 hours prior to planned study procedure

17. Active peptic ulcer or gastro-intestinal bleeding,

18. Active inflammation of the kidney interfering with diagnosis and treatment of RAS (e.g. glomerulonephritis, aortitis, vasculitis),

19. Radiation damage of the kidney,

20. Renal disease associated with aortic aneurysm i.e. diameter of the aorta > 40 mm,

21. Chronic renal replacement therapy,

22. Life expectancy < 1 year,

23. Co-morbid conditions limiting participation and follow-up,

##### Others

24. Patient currently participating in another trial possibly influencing the safety of the patient and/or the outcomes of the study,

25. Pregnancy/Planned pregnancy/Childbearing potential without sufficient measures to prevent pregnancy as judged by the investigator,

26. Known allergy to contrast medium that cannot be adequately controlled with pre-medication,

27. Known intolerance against acetylic-salicylic acid (ASA), heparin, clopidogrel and ticlopidin, cobalt-chromium,

28. Metformin intake not stopped at least 3 days before the intervention.

### Sample Size Calculation, Statistics, and Calculations

#### Sample Size Calculation

The primary endpoint is the difference between treatments in change of eGFR over 12 month. For this endpoint the intention-to-treat population (ITT) will be taken for primary analysis.

Definitions:

• Controls expectation value μ_C_, variance σ_C _^2 ^and group size n_C _= Medication

• Treatment expectation value μ_T_, variance σ_T _^2 ^and group size n_T _= Medication and Stent

A two-sided test will be performed.

• Null hypothesis: H_0_: μ_T _= μ_C_

• Alternative hypothesis: H_A_: μ_T _≅. μ_C _(↔.μ_T _< μ_C _or μ_T _> μ_C_)

• Minimum difference: Δ = μ_T _- μ_C_

• Variances: σ_T _^2 ^and σ_C _^2^

• Level of significance: α

• Statistical power: 1 - β

• Sample size: N = n_T _+ n_C_

##### Calculations

The sample size (N) has been calculated using the methods of Bock J, 1998 [22] and Chow

SC, 2003 [23], N = nT + nC. 4 (u1.α/2 + u1.β)2 (σ/Δ)2 for σC 2 = σT 2 = σ2

The probability for incorrect rejection of H0 for a two-sided test (α/2 = 0.025) is u1-0.025 = 1.9600.

The probability for correct rejection of H0 (1-β = 80%) is u0.8 = 0.8416.

Based on published data [24], the estimated GFR difference between baseline and 12 month follow-up in patients with unilateral RAS>70% is 4.35 ml/min. Moreover, from the DRASTIC study [25] considering the patient population with bilateral stenosis the estimated GFR difference at 12 months was 10.0 ± 16 ml/min. Furthermore, in a usual patient population the proportion of patients with bilateral RAS is 15% to 20%. This leads to a calculated increase of estimated ΔGFR for a mixed cohort of patients with unilateral and/or bilateral RAS of approximately: ΔeGFR = 0.2*10+0.8*4.35 ≈ 5.5 ml/min μ_T _= 5.5 ml/min.

In addition, for the medication group, we can assume an eGFR difference or decrease of

μ_C _= -1.0 ml/min at 12 months follow up.

The relevance level is assumed to be 20% of the difference between the therapy and medication or control group. Therefore, the sample size can be calculated as shown in Table 1.

**Table 1:** Calculation of sample size

**Expectation value**			**Relev**.	**Variance^1/2^**			**Sig. level**	**Statistical**	**Sample**
			
**Therapy**	**Ref**.	**Min. diff**.	**level**	**Therapy**	**Ref**.	**k **=	**1-sided**	**power**	**size**
**mue_T_**	**mue_C_**	**Delta**	**delta**	**sigma_T_**	**sigma_C_**	**n_T_/n_C_**	**alpha = 0.05**	**1-beta**	**N = n_T_+ n_C_**

5.5	-1.0	6.5	2.0	14.0	14.0	1.50	1	0.80	**250**

Considering a drop-out rate of approximately 20%, and a PTRAS rate of 20% of patients being initially randomised to BMT group, a total of 300 patients will be enrolled, 150 patients per group. A study patient that has been withdrawn from the study will not be replaced. It is intended to include between 6 and 24 patients per centre.

### Secondary endpoints

#### Improvement or stabilisation of renal function

The improvement or stabilisation of the renal function will be determined by change in eGFR at baseline/before start of treatment and 12 months after start of therapy. The following definitions will be used to assess the outcome on the renal function for comparison of the stent plus best medical therapy and the best medical therapy alone group [26]:

• Improvement: Increase of the absolute value of the eGFR after treatment by equal or more than 10% compared to the baseline value

• Stabilisation: Absolute value of the eGFR within ± 10% of the baseline value

• Failure: Decrease of the absolute value of the eGFR after treatment by equal or more than 10% compared to the baseline value.

GFR can be estimated by including serum creatinine with other demographic measurements in a prediction equation. These equations are valid only if the renal function is in a steady state, which can be defined by a constant serum creatinine in a given time interval. The eGFR will be calculated according to the following Modification of Diet in Renal Disease (MDRD) Study Prediction equation: eGFR (ml/min/1.73 m2) = 170 × [serum creatinine, mg/dL]^-0.999 ^× [age in years]^-0.176 ^× [0.762 if female] × [1.180 if black] × [serum urea nitrogen, mg/dL]^-0.17 ^× [serum albumin, g/dL]^+0.318^

#### Renal resistance index

Intra-renal Resistance Index (RI) will be calculated by the following formula: Intra-renal RI = (Peak Systolic Velocity-End Diastolic Velocity)/Peak Systolic Velocity

An intra-renal resistance index difference ΔRI > 0.05 is defined as haemodynamically relevant.

(≥ 70% diameter stenosis) in case of unilateral lesions, with the lower value on the side of stenosis.

#### Restenosis rate

Diagnosis of restenosis will be based on a colour-coded renal duplex sonography. A haemodynamically relevant unilateral restenosis (≥ 70%) is defined by a ΔRI more than 0.05. For bilateral lesions, haemodynamically relevant restenosis is defined by an acceleration time of more than 0.07 sec.

In case of haemodynamically relevant restenosis patients will be treated according to hospital practice.

#### Clinical events

Clinical events will be analysed for:

• Overall combined events (i.e. renal or cardiac death, stroke, myocardial infarction, hospitalisation for congestive heart failure, progressive renal insufficiency (i.e. need for dialysis), need for permanent renal replacement therapy, and need of TVR or TLR),

• renal clinical events (i.e.,. renal death defined as patient death classified according to the *International Classification of Disease*, *Tenth _Revision *(ICD-10) codes N00-N07, N17-

N19, and N25-N27, progressive renal insufficiency (i.e. need for dialysis), need for permanent renal replacement therapy, and need of TVR or TLR),

• MACCE (i.e. cardiac death, stroke, myocardial infarction, hospitalisation for congestive heart failure),

• individual events.

#### Determination of technical success

Technical success defined as successful access and deployment of the device with appropriate lesion coverage, stent positioning and patency determined by angiography at the time of deployment [27].

#### Procedural success

Acute procedural success defined as residual percent diameter stenosis <30% by quantitative computerised angiography [27]. Percent diameter stenosis is calculated as residual target lesion lumen divided by diameter reference segment. Procedural success defined as successful lesion crossing and stent positioning without the occurrence of a serious adverse events up to the moment the introducer sheath is removed [27].

#### Evaluation of blood pressure

Blood pressure will be monitored in every patient throughout the study as part of the basic medical care. In addition to that 24 hour ambulatory blood pressure will be recorded at randomisation, post procedure, at 2, 6 and 12 months (except post procedure for conservative group).

After completion of the study, average 24 h-blood pressure will be analysed and categorised according to the following definitions [34]:

• Cure: Diastolic blood pressure < 80 mm Hg and systolic blood pressure < 125 mmHg, off anti-hypertensive medications.

• Improvement: Diastolic blood pressure < 80 mmHg and/or systolic blood pressure < 125 mmHg on the same or reduced number of medications, or a reduction in diastolic blood pressure by at least 15 mmHg on the same or reduced number of medications.

• Failure: no change or inability to meet above criteria for cure or improvement.

• Benefit: cure or improvement.

#### Left ventricular mass index

Left ventricular mass index (LVMI) will be assessed by echocardiography or MR angiography at baseline and 12 months. LVMI (g/m^2^) is defined as the left ventricular mass divided by the body surface [m^2^]. Parameters for assessing myocardial muscle mass will be acquired by an experienced examiner according to the Guidelines of the American Society of Echocardiography. Therefore, myocardial muscle mass is calculated by using the method described by Devereux and Reichek, or as supplied by the manufacturer of the ECHO device. It has to be ensured that the same method is used in an individual patient.

#### Kidney size

The size of the kidney supplied by the renal artery that determined the patient's eligibility for the study will be measured by renal duplex sonography at baseline and after 6 and 12 months.

#### Evaluation of anti-hypertensive medication

All medication of the patients will be recorded in the CRFs and any changes in the medication must be recorded. Per patient antihypertensive medication will be analysed according to total number of anti-hypertensive medications, drug name, drug class, daily dose and regimen. Change in medical treatment will be compared with regard to anti-hypertensive medication using the classification of WHO daily defined doses (DDD). Total amount of anti-hypertensive medication recorded at different time points will be calculated according to the following formula:

Dose per day/DDD for this drug

Anti-hypertensive medication for each patient at baseline, 2, 6 and 12 months will be compared to the medication taken before randomisation.

#### Classification of heart failure

Heart failure will be classified according to the classification of the New York Heart Association at baseline, 2, 6 and 12 months.

#### Quality of Life assessment

During the study the quality of life will be assessed at baseline, 6 and 12 months using a validated questionnaire [28] (SF-12v2™) established to measure the impact of disease on the patients' quality of life. It has been used extensively as a screening tool, and is frequently used as part of longer, condition-specific surveys. Questionnaires will be supplied in the validated version of local language(s) of each participating country.

#### Patient survival

Patient survival will be calculated after the last follow-up has been completed as percentage of patients still alive and followed-up compared to treated/randomised patients.

#### Evaluation of safety

The safety of the Dynamic Renal stent will be analysed by adverse events and device related adverse events.

### Statistical Analyses

All patients will be analysed on an intent-to-treat basis. Data analysis will be performed on all patients who meet the eligibility requirements of this study using descriptive methods. Discrete variables will be presented as counts and percentage of the total. Continuous variables will be presented as mean ± standard deviation. In case of relative likelihoods the exact one-sided confidence interval of 95% will be calculated.

For the confirmatory analysis of relative likelihoods a binominal test will be performed. For the confirmatory analysis of metric data a t-Test will be performed. If a Kolmogorov-Smirnov-Test shows the data are not normally distributed, a non-parametric Wilcoxon-Test or in case of an inter-individual comparison of 2 datasets a Mann-Whitney-U-Test will be performed.

It is intended to perform the following sub analyses:

- Analysis by treated lesion,

- Analysis including time points after receipt of the Dynamic Renal stent during the study from patients initially randomised to the no revascularisation group.

Other subgroup analyses will be specified in the statistical analysis plan.

### Definition of Clinical Events

*Adverse Event Reporting*: An Adverse Event is any untoward medical occurrence in a clinical investigation patient, which does not necessarily have a causal relationship with this treatment. An adverse event (AE) can therefore be any unfavourable and unintended sign (including an abnormal laboratory finding), symptom, or disease temporally associated with the use of a medical (investigational) product, whether or not related to the medical (investigational) product. Significant device failure may constitute an adverse event if an undesirable experience occurs.

Adverse events information will be collected throughout the study in the Case Report Forms (CRF). Adverse events will be recorded on the CRF by the Investigator or a person authorised by the investigator. Event, date of onset, severity, duration, and relationship to device or prescribed drug regimen (see 5.7) will be recorded in the CRF. Any adverse events will be monitored until they are adequately resolved or reach a stable state.

#### Adverse Device Effect

An adverse device effect is any untoward and unintended response to a medical device. This includes any event resulting from insufficiencies or inadequacies in the instructions for use or the deployment of the device. Also user errors that result in an untoward and unintended response are included. Adverse device effects information will be documented in the CRF as adverse events related to the device.

#### Serious Adverse Event

Serious adverse events are adverse events that

a) led to death,

b) led to a serious deterioration in the health of the subject that

1) resulted in a life-threatening illness or injury,

2) resulted in a permanent impairment of a body structure or a body function,

3) required in-patient hospitalisation or prolongation of existing hospitalisation,

4) resulted in medical or surgical intervention to prevent permanent impairment to

body structure or a body function.

c) led to foetal distress, foetal death or a congenital abnormality or birth defect.

The Investigator must judge on whether each event meets the definition of a "serious" adverse event. All serious adverse events and deaths must be reported by fax to BIOTRONIK AG within 24 hours after the investigator learns of the event using the SAE report forms. These are provided to the investigator together with the CRFs.

#### Serious Adverse Device Effect

Adverse device effect that has resulted in any of the consequences characteristic of a serious adverse event or that might have led to any of these consequences if suitable action had not been taken or intervention had not been made or if circumstances had been less opportune. Serious adverse device effects (SADE) must be reported by fax to BIOTRONIK AG within 24 hours after the investigator learns of the event using the SAE report forms. These are provided to the investigator together with the CRFs.

#### Regulatory Reporting

Investigators are responsible for reporting SAEs and SADEs to their reviewing Independent Ethic Committee (IEC)/Institutional Review Board (IRB) according to the local applicable regulations.

#### Criteria of Judgment on Relationship to Treatment

All adverse and serious adverse events have to be judged by the investigator for their relation to the study treatment by help of the following criteria: timely relation of the event to the procedure, known risks of the device and intervention, concomitant diseases, concomitant measures and medication, other possible explanations, as applicable. Causality to the treatment will be judged using the following categories: not related, possibly related, probably related, related. No other category applies. Documentation about judgment and causality assessment has to be available for review and be provided upon request.

### Study device

The BIOTRONIK Dynamic Renal stent system incorporates the latest technical developments of approved state-of-the art renal stent systems. The safety and performance of these reference devices was demonstrated in clinical studies which together included more than 260 procedures. Dynamic Renal is equivalent to the reference stent systems (Palmaz Blue from Cordis Corporation a Johnson & Johnson company and Herculink Elite from Abbott Vascular Corporation) due to its very similar design, materials and technical characteristics. Although the clinical behaviour of a stent system can not be entirely predicted from its technical characteristics or its similarity with established stent systems, it is justified to expect that the Dynamic Renal stent system will be equivalent to the marketed predecessor and reference devices during clinical use.

The Dynamic Renal stent is a tubular, balloon-expandable stent sculpted by laser from a single tube of L-605 cobalt-chromium (CoCr) alloy. The stent consists of circular segments at each end followed by a transition zone and struts arranged in a double helix in the middle. Each loop of the helices is connected to the next loop by 2–3 (alternating) longitudinal struts. This design is broadly similar to that of the approved Dynamic stent system [29]. The stent surface is fully coated with a layer of amorphous hydrogen-rich silicon carbide (a-SiC:H). This material has semi-conducting properties that efficiently prevent the electron transfer from fibrinogen to the metal surface in vitro. Thereby the conversion from fibrinogen to fibrin and it's deposition at the stent surface is reduced [30]. Additionally, a-SiC:H-coated stents exhibit a lower adhesion and activation of blood platelets and leucocytes [31]. Finally, the release of potentially allergenic ions from a silicon carbide coated stent is substantially reduced in comparison to an uncoated metal stent. This coating is used on all BIOTRONIK coronary and peripheral stents produced so far and was evaluated in a number of clinical studies [6,32-36]. Full information can be deduced from the applicable instructions for use.

The stent delivery system is based on a rapid-exchange balloon catheter. The stent is securely crimped on a polyamide balloon situated at the distal tip of the catheter between two radiopaque markers made of a Platinum-Iridium alloy. The proximal shaft of the delivery system consists of a polyamide tube (PA12) that incorporates a stiffening wire made of stainless steel (AISI 301) to improve its pushability. It has a single Luer port for connecting an inflation/deflation device to inflate/deflate the balloon. The distal section of the catheter comprises the inflation/deflation (balloon) lumen and the 15 cm long guide wire lumen which starts at the catheter tip and ends at the guide wire exit. It accepts guide wires of 0.014" diameter. The stent delivery system is compatible with guiding catheters with a minimal inner diameter of 0.070 inch (1.78 mm, 6F sheath compatible) or introducer sheaths of 4 to 5 F.

#### Device sizes

The Dynamic Renal stent system is available in 16 different variants. The stent is available in lengths of 12 and 19 mm. It is pre-mounted on a delivery system which has nominal balloon diameters of 4.5, 5.0, 6.0 and 7.0 mm and usable lengths of 80 cm and 140 cm respectively that allow a femoral or brachial/radial access route.

### Study Committees

#### Data and Safety Monitoring Board (DSMB)/Clinical Events Committee (CEC)

In this study, the DSMB/CEC is composed of five members (four physicians with sufficient experience in the targeted indication, and one biostatistician), who are independent of the conduct of the trial.

Based on the safety data, the DSMB/CEC may recommend to modify or stop the trial. The DSMB/CEC will discuss and classify all clinical events for later analysis. At the onset of the trial, the DSMB/CEC established explicit rules outlining the minimum amount of data required, and the algorithm followed in order to classify a clinical event. BIOTRONIK will compile clinical event packets when the necessary data are available from the investigative sites. They will provide this information to the DSMB/CEC. The DSMB/CEC will meet based on event rate accrual to review and adjudicate all clinical events for which the required minimum data is available. The Committees will also review and rule on all deaths that occur throughout the trial.

#### Coordinating and Co-Coordinating Investigators

Thomas Zeller, MD, Department Angiology, Herz-Zentrum Bad Krozingen, Südring 15, 79189 Bad Krozingen, Germany,

Marco V. Wainstein, MD PhD, Hospital de Clinicas de Porto Alegre, Serviço de Cardiologia

Sala 2059, Rua Ramiro Barcelos, 2350, Porto Alegre, RS 90035-003, Brazil

#### Sponsor of the study

BIOTRONIK AG, Ackerstrasse 6, 8180 Bülach, Switzerland

## Discussion

The RADAR study is the first randomised controlled trial on the impact of RAS compared to best medical therapy based on a strict physiological definition of the main inclusion criterion, the degree of stenosis. The lumen reduction of the lesion must result in a measurable reduction of kidney perfusion distal to the stenosis expressed by a significant reduction of the resistance index of the affected kidney. This reduction of the resistance index nicely correlates to an at least 70% diameter reduction by angiography [37,38]. With this strict inclusion criterion it is intended to randomise only patients in whom restoration of normal kidney perfusion should have an impact on kidney function and with this by modulating the renal hypoperfusion triggered humoral disorders also an impact of secondarily affected organs such as the heart and the brain.

All former comparative studies on RAS were more liberal with their inclusion criteria accepting even moderate degrees of RAS, e.g. DRASTIC included about one third of patients having a degree of angiographic diameter stenosis less than 70% [12]. Even the recently at the Annual Meeting of the American College of Cardiology (Philip Kalra) presented so far largest study including more than 800 patients, the ASTRL trial, enrolled 25% of the patients with RAS less than 70% (range 20–100%) diameter stenosis. Due to the inclusion of patients without haemodynamically relevant RAS and the small sample sizes (DRASTIC 102 patients), all currently published studies failed in proving a significant benefit of renal artery intervention on blood pressure control and renal function [12-14]. Noteworthy GFR in the DRASTIC study increased by 15 ml/min in the balloon angioplasty group whereas it slightly decreased by 1 ml/min in the control arm. However, the study was not powered adequately to show a significant difference. The sample size calculation for the present study was based on a recently published pilot study (PRECISON [39]) for the trial and the so far reported controlled trial data. Enrolling in total 300 patients in a 1:1 randomised fashion we expect a significant difference in the primary study endpoint, the difference between treatments in change of eGFR over 12 months.

Renal function is one of the most powerful predictors of survival [40]. Another one is left ventricular hypertrophy [41]; the analysis of the course of left ventricular mass index is also included as a secondary endpoint because the reducing the activation of the renin-angiotensin-aldosterone-system by RAS revascularisation should lead to a regression of left ventricular hypertrophy [41]. Affecting these major endpoints, renal function and left ventricular hypertrophy, RAS stenting should also impact the composite clinical endpoint of patient survival, hospitalisation and end-stage renal failure after 3 years.

Provided a timely initiation of all study sites it is expected to finish patient enrolment within 1 year so that the analysis of the primary endpoint should be available in 2011.

## Conclusion

Based on the strict physiological definition based on reduction of the resistance index, including only patients with haemodynamically relevant RAS and on enrolling in total 300 patients in a 1:1 randomised fashion the expected outcome of the study is a significant difference in the primary study endpoint, the difference between treatments in change of eGFR over 12 months.

## Abbreviations

": Inch; 24h-BP: blood pressure obtained during 24 hour continuous assessment; ADE: Adverse device effect; AE: Adverse event; ASA: acetylic salicylic acid; a-SiC:H: amorphous hydrogen-rich silicon carbide; BMT: Best Medical Treatment; BP: blood pressure; CEC: Clinical Event Committee; cm: centimetre; CoCr: Cobalt chromium; CRF: Case report form; CT: Computerized tomography; DDD: defined daily dose according to WHO drug dictionary; dL: decilitre; ΔRI: difference in RI between the two kidneys; DSMB: Data Safety Monitoring Board; ECHO: Echocardiogram; eGFR: estimated GFR; g: gram; F: French; g/m^2^: gram per square meter; GFR: glomerular filtration rate (in this study the eGFR will be used); HbA1c: hemoglobin A1c; ICD: International Classification of Disease; i.e.: *id est *(that is); IEC: Independent Ethic Committee; INR: International normalized ratio; IRB: Institutional Review Board; ITT: intention to treat; LDL: low density lipoprotein; LVMI: left ventricular mass index; m^2^: square meter; MACCE: Major Adverse Cardiac and Cerebral Events; MDRD: Modification of diet in Renal Disease (study); mg: milligram; min: minute; ml: millilitre; mm: millimetre; mmHg: Milliliters of mercury; MR: Magnetic resonance; NYHA: New York heart association; PAD: peripheral artery disease; PTRA: percutaneous transluminal renal angioplasty; PTRAS: percutaneous transluminal renal arterial stenting; RAS: renal artery stenosis; RCT: Randomised controlled trial; RDS: renal duplex sonography; RI: resistance index; RVD: reference vessel diameter; SADE: Serious adverse device effect; SAE: Serious adverse event; SCr: serum creatinine; sec: Second; TLR: target lesion revascularization; TVR: target vessel revascularization; U: Unit; WHO: World Health Organization.

## Competing interests

The authors declare that they have no competing interests.

## Authors' contributions

The draft of the manuscript was prepared by Uwe Schwarzwälder and Michael Hauk and finally approved by Thomas Zeller.
